# Programming temporal morphing of self-actuated shells

**DOI:** 10.1038/s41467-019-14015-2

**Published:** 2020-01-13

**Authors:** Ruslan Guseinov, Connor McMahan, Jesús Pérez, Chiara Daraio, Bernd Bickel

**Affiliations:** 1 Institute of Science and Technology Austria, Am Campus 1, Klosterneuburg, 3400 Austria; 2 California Institute of Technology, 1200 E California Blvd, Pasadena, CA 91125 USA; 3 Universidad Rey Juan Carlos, Calle Tulipán, s/n, Móstoles, 28933 Madrid Spain

**Keywords:** Mechanical engineering, Polymers, Design, synthesis and processing

## Abstract

Advances in shape-morphing materials, such as hydrogels, shape-memory polymers and light-responsive polymers have enabled prescribing self-directed deformations of initially flat geometries. However, most proposed solutions evolve towards a target geometry without considering time-dependent actuation paths. To achieve more complex geometries and avoid self-collisions, it is critical to encode a spatial and temporal shape evolution within the initially flat shell. Recent realizations of time-dependent morphing are limited to the actuation of few, discrete hinges and cannot form doubly curved surfaces. Here, we demonstrate a method for encoding temporal shape evolution in architected shells that assume complex shapes and doubly curved geometries. The shells are non-periodic tessellations of pre-stressed contractile unit cells that soften in water at rates prescribed locally by mesostructure geometry. The ensuing midplane contraction is coupled to the formation of encoded curvatures. We propose an inverse design tool based on a data-driven model for unit cells’ temporal responses.

## Abstract (web summary)

Shape-morphing materials from initially flat to curved surfaces don’t tend to account for the rates of shape transformation. Here, the authors develop an algorithm for design and fabrication of plates that morph in water taking the desired 3D shape with deformation rates specified across the surface.

## Introduction

Morphing flat sheets into complex, three-dimensional geometries is a challenge that has been pursued for centuries by artists, and more recently by mathematicians and scientists^1–3^. In engineering, the search for materials suitable for such transformations has been motivated by the ease of two-dimensional fabrication^4^, which relies on subtractive processes, such as punching, machining, water jetting, or laser cutting. Flat objects can be stacked in volumetrically efficient arrangements, which simplifies transportation and storage. While flat sheets are easy to fabricate and store, many structural and functional applications across scales rely on changing surface curvatures (e.g., tunable mirrors^5,6^ and parabolic antennae^7^). Morphing between flat geometries and desired curved surfaces requires methods for prescribing local deformations.

To reach non-zero Gaussian curvatures from initially flat shells, bending must be coupled to in-plane stretches, according to Gauss’ *Theorema Egregium*^8^. Several frameworks have been proposed to achieve this. Notably, the out-of-plane deformations of auxetic and kirigami sheets are defined by the architecture of voids or cut patterns^9–11^. Kinematic frustration has recently been embraced for changing the curvature of initially flat shells^12,13^. These examples are suitable for lightweight structures, but require mechanical stimuli to achieve 3D shapes through manual forming, boundary loading, or through the release of a pre-stretched sheet. Self-actuation is desirable in morphing shells because it enables untethered structural adaptation to changing environmental stimuli. To this end, responsive materials combine structural, sensing, and actuation capabilities in structures that remain flat until a non-mechanical environmental stimulus triggers the actuation process.

For example, self-actuation has been demonstrated in shells through hydrogel swelling^2,14^ and nematic-to-isotropic phase changes in liquid-crystal elastomers^15^. A variety of 4D-printed systems can also be used to achieve desired shapes by coupling locally prescribed in-plane kinematics to changes in curvature^16–22^. Self-actuation has been extended to the folding of origami^23,24^, which is one of the most common and well-studied methods for inducing shape changes in initially flat objects^3,4,25,26^. However, none of these proposed solutions demonstrate control over deformation rates during morphing processes. Consequently, self-collisions may occur in attempts to realize more complex geometries.

The ability to locally control the shape evolution in time drastically expands the design space of self-morphing shells. More broadly, an intrinsic capacity for pre-programmed temporal responses allows designing materials that can perform complex tasks, like self-deployment and locomotion, without the need for external controllers or power supplies^27^. While time-dependent folding has been demonstrated in structures that are wired to power sources and electronic control devices^28,29^, a small number of architected shells made of materials with intrinsic actuation capabilities have incorporated temporal programming through the sequential folding of discrete hinges^30–32^. However, none of these examples allow for changes in Gaussian curvature. They realize sequential folding of few discrete hinges^30^, must be fabricated in their target shape prior to manual programming^31^, or rely on ad hoc empirical designs that do not account for characterizations of their materials’ time-dependent constitutive responses^32^.

Here, we show that spatio-temporal information can be embedded in the geometry of architected shells that morph from flat to smooth three-dimensional shapes. This programmed temporal evolution enables reaching target geometries that would be impeded by collisions if shells actuated with uniform or unplanned deformation rates. Furthermore, our shells use polymers that actuate when the temperature in their environment is set to a critical value. At room temperature, they remain flat, storing the energy necessary to drive the deformation.

## Results

### Spatio-temporal programming of self-actuated shell mechanisms

**Fig. 1:**
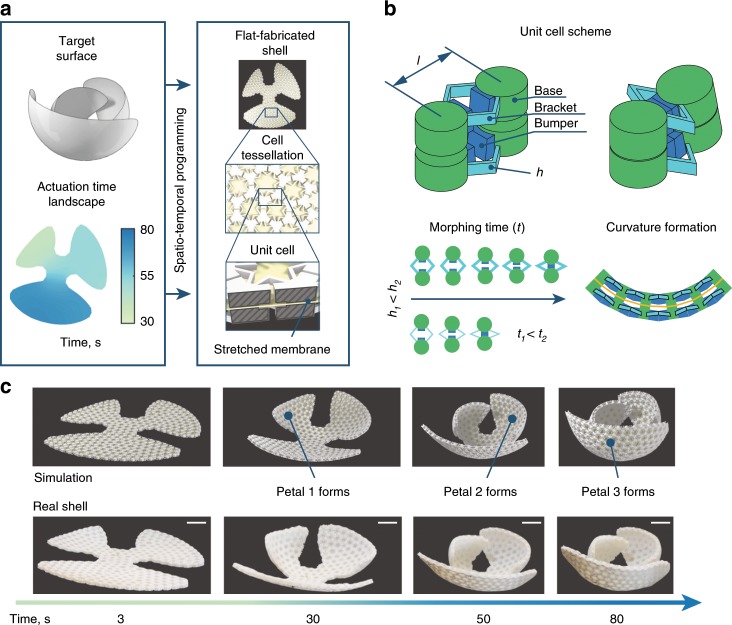
Encoding spatial and temporal shape evolution in a flat shell mesostructure. **a** A user-specified target surface and actuation time landscape (a field of deformation completion times) are inputs to an inverse design procedure that defines the mesostructure of flat-fabricated shells that morph into the target geometries. The shells are composed of inhomogeneous tessellations of unit cells with an interior pre-stretched membrane. **b** Each unit cell has an initial central length *l*. Brackets control actuation time through their softening rate, which is controlled by their thickness, *h*, and a set of bumpers prescribe final local curvatures upon collision. **c** Morphing of a petalled structure with an actuation time landscape ensuring that larger petals cover their smaller neighbors avoiding collisions on the way. Simulation and experiments are compared at 3, 30, 50, and 80 seconds in water. The structure replicates the encoded actuation time landscape shown in (**a**). Scale bars, 3 cm.

We propose an inverse design algorithm for shell architectures and the temporal evolution of their shapes (Fig. 1a). The algorithm collects user inputs at two stages: the first input is the desired 3D target surface and the second is the specification of local deformation rates. We term this temporal map input an *actuation time landscape*. The algorithm outputs the mesostructure for initially flat shells that we fabricate and test. These shells have three layers, with a $\sim \! 4.6\ {\rm{mm}}$ total thickness. The two outer layers are 3D-printed tessellations of non-uniform unit cells, made of Vero PureWhite (Stratasys). The middle layer is a $0.5\ {\rm{mm}}$ thick pre-stretched elastic membrane, which stores the energy required to drive the morphing process. Actuation from the flat to the curved profile is triggered by immersing the shells into 56 °C water, which causes the outer layers to soften over the course of approximately 30–80 s.

The unit cells have a grid spacing of $\sim \!\! 10\ {\rm{mm}}$, and are composed of cylindrical bases connected at the external shell surfaces by pairs of V-shaped brackets (Fig. 1b). The bases serve as attachment points to the elastic membrane and as mounting points for the brackets. The brackets serve as nonlinear springs: they hold the structure flat prior to being placed in water, and then they guide the temporal morphing process, softening at rates determined by their geometric parameters when heated (thicker brackets soften at slower rates). There are also bumpers attached to the bases in the space between the brackets, which collide when the local contraction reaches the target magnitude and control the contact angle between adjacent unit cells. The membrane provides energy for actuation, compressing the brackets as they soften. All of these components play an important role for reaching targeted geometries through spatio-temporal programming. For example, the petalled structure shown in Fig. 1c has been programmed so that the petals reach their target shape sequentially, actuating from smallest to largest (Supplementary Movie 1). If all petals deformed at the same rate, they would collide and would not reach the target geometry. More details on shell design are available in the Supplementary Note 1.

We incorporated a discretized mechanical model of our shells in an inverse design algorithm for obtaining desired temporal morphing. Given a target geometry and a smooth time landscape, the algorithm automatically generates the flat shell mesostructure that will produce the corresponding morphing process. It first composes a continuous target shell out of compressed unit cells. To do this, the target surface is isotropically triangulated, producing a stencil that serves as a placeholder for base locations. With the arrangement of bases in the target shape, the bumper geometries are defined to ensure that they are in contact in this target shape (Fig. 1b top-right). Then, a minimal distortion conformal map^33^ flattens the stencil. The bases with bumpers are then relocated to the flat stencil and are interconnected by brackets. Note that this stencil has to be free of overlaps to enable fabrication. Therefore, base placement, bumper arrangements, and bracket lengths are configured automatically given a target surface input. However, the selection of bracket thicknesses is governed by the designer’s specification of the actuation time landscape. Thicker brackets soften at a slower rate than narrower ones, enabling distinct target deformation times to be realized in each region of the shell for collision avoidance, visual impression or other desired functionalities. Given that there can be a broad range of morphing sequences that yield certain target geometries, the morphing process can be designed according to the designer’s goals by iterating through actuation time landscapes and observing their effect.

The time evolution of the shells is simulated quasistatically by coupling a finite element simulation of the rubber membrane with a data-driven spring model for the brackets and a rigid body model for bases. Bumper collisions are described as sharp increases in bracket stiffness in the model. A summary of the energy model is given below. Its constitutive parts are the energy associated with bracket compression (${W}_{{\rm{c}}}$) obtained from fitting and interpolating experimental data, an energy penalty to shearing (${W}_{{\rm{s}}}$) that replicates the effect of the shear-resisting bracket geometry, and the elastic membrane energy (${W}_{{\rm{m}}}$):

1 $$W({\bf{x}})=\mathop{\sum }_{{c}_{ij}}{W}_{{\rm{c}}}+\mathop{\sum }_{{s}_{ij}}{W}_{{\rm{s}}}+\mathop{\sum }_{{{\mathcal{T}}}_{i}}{W}_{{\rm{m}}}({{\bf{G}}}_{i}).$$

Here, ${c}_{ij}$ refers to the contractile springs that join the $i$th and the $j$th bases. Each unit cell is modeled with four of these springs to capture bending effects. Bumper collisions are modeled as a sharp stiffening of these elements. Shear-resisting elements ${s}_{ij}$ have analogous indexing. ${{\mathcal{T}}}_{i}$ refers to the $i$th element of the membrane discretization, and ${{\bf{G}}}_{i}$ is the deformation gradient of the membrane evaluated at this element. A complete description of the simulation procedure is available in the Supplementary Note 2.

**Fig. 2:**
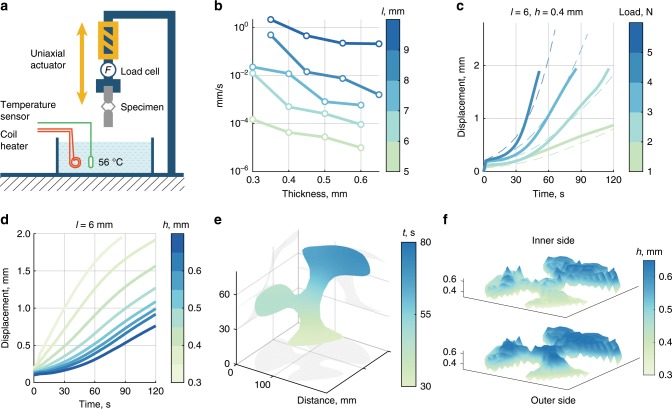
From measurement of brackets’ mechanical properties to inverse design of temporal morphing. **a** Load-controlled tensile tests were used to determine the deformation rates of unit cells in 56${}^{\circ }$ C water. **b** Average deformation rates for specimens subject to constant loads of 4 N for *l*$\, < \,7$ and 5 N for *l*$\, \ge$ 7 N. These values are close to the inner membrane tractions on each unit cell in real shells. **c** Deformation rate measurements (solid lines) are fit (dashed lines) to produce a model of bracket softening. Here we show the fit for *l* = 6 mm, *h* = 0.4 mm. **d** The model is interpolated and queried to infer the mesostructure that yields target curvatures and deformation completion times in each section of the shell. Here, we show deformations of unit cells with central length *l* = 6 mm and a range of bracket thicknesses from 0.3 mm to 0.65 mm. **e** Smooth actuation time landscape that induces the sequential deformation process demonstrated in Fig. 1. **f** Bracket thickness fields for both sides of the petalled shape. Though the prescribed time landscape is smooth, the field of bracket thickness is highly irregular because bracket thicknesses also depend on initial unit cell lengths and their target deformations.

To construct the constitutive model for bracket softening, we conducted experiments (Fig. 2a) on brackets of varying length $l$ (in a range 5–9 mm) and thickness $h$ (0.3–0.65 mm), applying constant forces (1–5 N) and tracking their compression over time spent in water using a Zwick tensile tester (Figs. 2b, c). Fits to the experimental data were then sampled from the space of bracket parameters and immersion times to build a time-dependent force–displacement model used in the simulation (Fig. 2d). Material measurement and modeling are discussed more extensively in the Supplementary Note 3. From this sampling, we select bracket thicknesses that yield target deformation timings under the loads generated by the membrane (see Supplementary Note 4).

### Examples of temporally programmed structures

**Fig. 3:**
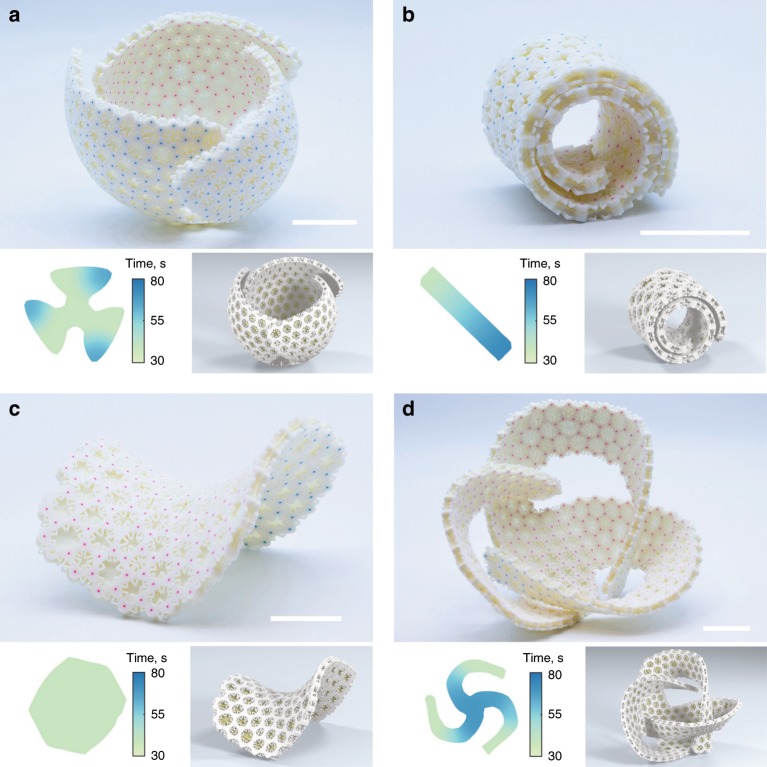
Spatio-temporally programmed shells. Each panel shows a real shell (top), its actuation time landscape (bottom-left), and its corresponding simulated shell (bottom-right). **a** Doubly curved shell where petals morph synchronously to cover each other in a cyclic manner. One corner of each petal is programmed to morph slower to increase the distance between petals during morphing. **b** A double spiral that approximates a developable surface. A gradient time landscape enables the inner spiral to curl first. **c** A saddle shape with negative curvature. **d** A shell with a complex self-interweaving shape prone to multiple collisions in the course of its morphing process. Scale bars, 3 cm.

We highlight the effect that different actuation time landscapes have on the final shapes of initially flat shells by comparing the example discussed in Fig. 1c to the shell shown in Fig. 3a. Both shells have similar flat geometries but different actuation time landscapes are encoded in their mesostructures. In the first example, smaller petals are covered by their larger neighbors. Meanwhile, each petal shown in the second example has an edge that covers a neighbor and one edge that is covered. For the latter case, all the petals have been programmed with the same actuation time landscape so they deform simultaneously. We slightly increased target actuation times for some petal tips to increase the distance between neighboring petals on their morphing paths (Supplementary Movie 2). This way, the interior edge of each petal completes its deformation before being covered by its neighbor. Shape-morphing precision was measured using a 3D scan of the final geometry. The blue markers were matched with their simulated locations. The resulting mean error for the pairwise distances in the experimental realization of the structure shown in Fig. 3a was $3.6 \%$ relative to the diameter of the target markers’ point cloud.

Developable target surfaces can also be achieved using this structural framework. The actuation time landscape for the double-loop spiral shown in Fig. 3b is a constant gradient from one end to the other. This allows the interior to curl without colliding with the outer loop (Supplementary Movie 3). The 3D scan reveals a 2.4% mean error. Geometries with negative Gaussian curvature can also be realized, such as the saddle shown in Fig. 3c (Supplementary Movie 4). The relative mean error for the saddle’s base positions was $0.8 \%$.

We showcase the complexity of achievable shapes through the self-interweaving structure shown in Fig. 3d. This shape requires embedding precise morphing trajectories and time landscapes in the flat-printed structure in order to thread each arm through the loop created by a neighboring one without colliding (Supplementary Movie 5). The experimental replication of this challenging target geometry yields a 9.7% 3D scan mean error, and highlights the versatility of our design and simulation framework.

## Discussion

**Fig. 4:**
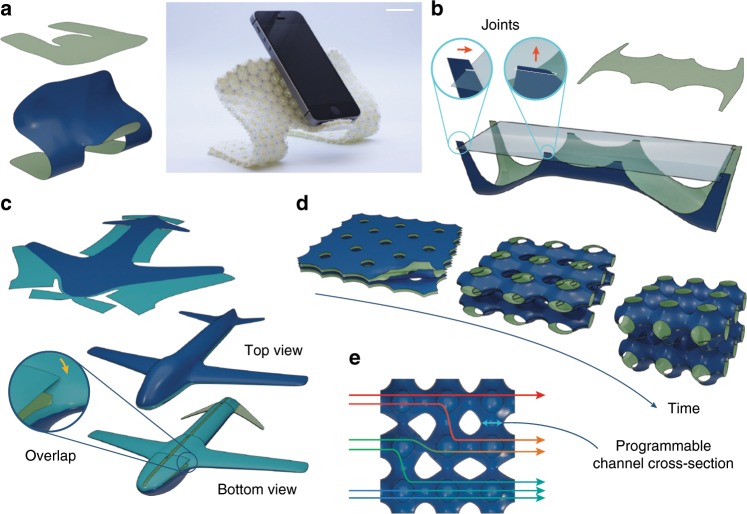
Future concepts for spatio-temporally programmed materials. Potential applications for shells with programmable self-morphing, showing initial flat and final states. Opposite sides of each sheet are colored blue and green. **a** The load-bearing capability of our morphing shells is shown by a mobile phone stand. Scale bar, 3 cm. **b** Self-morphing can be applied to industrial design, for example to build aesthetically curved furniture with sequentially interlocking joints. **c** The outer shell of a self-morphing drone can be fabricated from a single sheet. The smooth aerodynamic shape is achieved with temporal programming of sequentially overlapping parts. **d** Stacked multilayer sheets can be used to create stiff, yet lightweight periodic mesostructures with temporally programmed porosity. Four sheet layers (left) morph into a Schwarz P surface (right) which efficiently distributes external stresses through the structure. **e** Similar structures with programmable channel cross-section between the periodic blocks can be used to adjust flow rates through the device.

The realization of complex 3D geometries from flat-fabricated structures, which are easier to manufacture and transport, is a compelling motivation for developing shape-morphing frameworks. However, to be used in a broad range of engineering applications, the morphed structures must remain structurally stable. In our examples, the shells’ outer layers soften during deformation in hot water, but become stiff when returned to room temperature (see the details on the shells’ fabrication procedure in the Supplementary Note 5). We show their load-bearing ability realizing a mobile phone stand (Fig. 4a). Note that the mass of the phone (113 g) exceeds the mass of the shell (55 g). Data from simple mechanical tests on flat structures are available in Supplementary Note 6. Structural stability could be further increased by using snap-locking mechanisms instead of bumpers or coating the structure after actuation has completed.

Temporally programmed self-morphing opens the door to a broad range of industrial design applications. Sequentially interlocking joints, for example, can be employed in the self-assembly of initially flat furniture (Fig. 4b), while doubly curved surfaces can be used in drone shells (Fig. 4c). The Schwarz P triply periodic minimal surface structure (Fig. 4d) can be obtained from layered sheets that are programmed to have time-evolving porosity. Such structure could be used as an energy absorbing material or a microfluidic device with programmable channel cross-sections (Fig. 4e).

Our method for programming temporal morphing in shells is based on a combination of a pre-stressed midplane and outer layers with effective stiffness differentials that are configured to evolve over time according to user specifications. The encodement is performed using an inverse design algorithm that takes a target surface and an actuation time landscape as inputs and outputs a mesostructure with embedded self-morphing information. The significance of this method is that it enables collision-avoidance during deformations from flat shapes to curved geometries. We built a design system based on a data-driven mechanical model of mesostructures to predict shape evolution in time, enabling temporal morphing design. Applications of self-actuating shells to biomedical and construction industries are close to becoming reality with the fast advances in this field of study. Further generalizations of our approach to other materials such as liquid-crystal elastomers, bio-compatible polymers, and conventional engineering materials whose properties evolve in time due to other stimuli such as temperature, humidity, light, pH, etc., could enable rapid manufacturing of load-bearing structures that can only assume desired geometries through temporally planned deformations upon deployment as well as robotic materials temporally programmed for a broader range of functionalities.

## Methods

### Experiments

All specimens used for bracket characterization and the outer layers of the shape-morphing structures were 3D-printed using a Stratasys J750 using Vero Pure White material. Colored markers were included in the bases to facilitate visualization. Water was kept at 56 °C using a temperature controller, and two Canon 700D cameras were used for imaging.

The mechanical properties of the brackets were measured using a Zwick tensile tester with a custom-built water tank attachment. Experiments measuring strain restitution after unloading were conducted to estimate the plastic fraction of the deformation. These experiments are discussed more extensively in the Supplementary Information.

All shells were fabricated by first uniformly stretching a latex sheet of thickness 0.5 mm to 900% its initial area. After cleaning the membrane surfaces with 2-Propanol, the printed lattices are glued to the membrane. In each structure, several bases have holes to align the opposite shell layers using push-pins. Latex surplus surrounding the assembled flat shell is removed, then the shells are submerged into a $350\times 350\times 350$ mm water tank to induce shape-morphing. See the Supplementary Information for more details.

### Data fitting and simulation

Experimental data was used to generate a force-displacement model of brackets by combining simpler fitting components. First, displacement-force curves were fitted so that the initial state corresponded to the behavior at room temperature. Second, displacement rates dependent on time in water were fitted. Based on the combination of these two fittings, the parameter domain was resampled to yield time-dependent force-displacement relationships. Data from the plasticity experiments were used to build the final elastoplastic model used in the simulation software for inverse design.

In addition to implementing this data-driven bracket compression model in simulations, shear-resisting elements are included to capture brackets’ geometry-based shear resistance. Bases are simulated as rigid bodies and a FEM approach is used for the membrane simulation. Bumper collisions are modeled as abrupt bracket stiffness increases. All simulation elements are coupled via shared vertices. We implemented the simulations using a C++ code developed in-house. A simple user interface was designed to import target surfaces and specify time landscapes. User inputs are automatically processed to configure the simulated structure and display resulting morphing processes. Once the desired morphing is achieved, the system automatically generates structures for 3D printing.

The data-fitting and simulation procedures are explained in more detail in the Supplementary Materials.

### 3D scanning

An HP David SLS-3 structured light scanner with two cameras was used to generate textured 3D meshes in OBJ format. Then, the textures were filtered to obtain binary images with markers. Markers were lifted to their actual scanned positions using the 3D mesh and resolved to single points. The obtained point clouds were registered to the clouds generated by the simulation software and points were matched using Munkres’ algorithm.

## Supplementary information

Supplementary Information

Peer Review File

Description of Additional Supplementary Files

Supplementary Movie 1

Supplementary Movie 2

Supplementary Movie 3

Supplementary Movie 4

Supplementary Movie 5

## Data Availability

All raw data is publicly available through IST Austria Research Explorer 10.15479/AT:ISTA:7154.

## Code availability

Source code and processed data are publicly available on https://github.com/russelmann/temporal-morphing-ncomms.
