# Supplementary material for: Programming temporal morphing of self-actuated shells
Source: Nat Commun. 2020 Jan 13;11:237. doi: 10.1038/s41467-019-14015-2 (PMC6957700; doi:10.1038/s41467-019-14015-2)
Supplement: Supplementary file 1 — Supplementary Information [file 41467_2019_14015_MOESM1_ESM.pdf]

SUPPLEMENTARY INFORMATION

Programming temporal morphing of self-actuated shells

Guseinov et al.

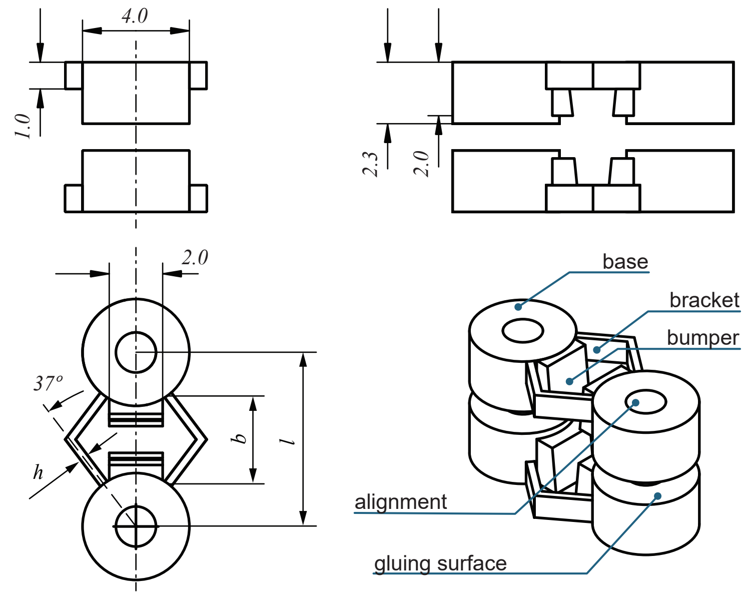

Supplementary Figure 1: Unit cell scheme. The configurable parameters are the central length l (constant difference with bracket length b), bracket thicknesses h (which can be different for the two opposite layers), and the bumper cutting plane.

Supplementary Note 1: Shell design

Our shells have three layers. Two 3D-printed non-uniform tessellations form the outer layers. These are glued to either side of a pre-stretched latex membrane. This section describes the design of the flat-printed geometries.

Single unit cell design

The shells' outer layers are tessellations of the structures shown in the Supplementary Figure 1. Each of these unit cells is bounded by four cylinders of diameter 4 mm and height 2.3 mm, called bases. The bases are connected to their neighbors by two symmetric V-shaped spring elements (called brackets) that form an angle of 37° relative to the central axis and have a height of 1 mm. At room temperature, brackets are sufficiently stiff to prevent finite deformations due to compression by the pre-stretched elastic membrane that constitutes the shells' mid-planes. The brackets soften when placed in hot water, inducing in-plane contraction. This contraction occurs until the bumpers attached to the bases in the space between the brackets collide. This collision occurs in each unit cell once the target in-plane deformation for that unit is reached. Local curvatures are programmed in a unit cell by setting different bumper lengths for the opposite outer layers. To facilitate aligning the two outer layers with respect to each other during shell fabrication, cylindrical holes are subtracted from several bases (see the Supplementary Note 5 for more information).

The feature dimensions were chosen for the following reasons. Bases interface the membrane to the brackets, so they must have a sufficiently large gluing area to be reliably connected to the membrane, but should be small enough to allow large curvatures in decimeter-scaled specimens. The bracket shape is designed to reduce both in-plane and out-of-plane shearing. In-plane shearing is prevented due to the large (37°) angle between the bracket and the central axis. Out-of-plane shearing is prevented due to the rectangular shape of the bracket section. The 1 mm bracket height is always larger than its thickness ($h \leq 0.65$ mm), which makes in-plane bending energetically favorable.

Tessellating unit cells

The design pipeline starts with a user-provided target surface (Supplementary Figure 2), which is isotropically remeshed into a triangular mesh $\mathcal{T}$ with a target number of vertices N [1]. Each pair of adjacent triangles of this mesh represents one unit cell with two bases placed on opposite faces of each triangle, centered on its

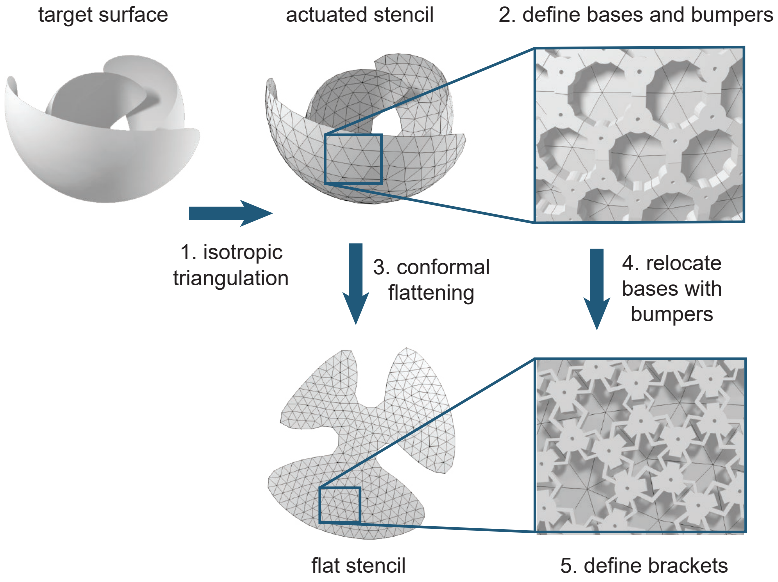

Supplementary Figure 2: Shell design pipeline: 1. A target surface is isotropically triangulated. 2. This "actuated" stencil is populated with bases and bumpers touching their corresponding neighbors. 3. The "actuated" stencil is conformally flattened. 4. Bases with bumpers are relocated to the flat stencil. 5. Bracket lengths are set by the distance between bases in this configuration. Bracket thickness is defined later during the temporal programming phase.

barycenter. Thus, the whole mesh $\mathcal{T}$ serves as a stencil for our structure's final state, when deformation has completed. It is scaled to ensure there is a minimal bumper length (0.3 mm) to limit bracket deformations in the final state. The actual size of the shell depends on the number of vertices in $\mathcal{T}$.

In order to generate a shell of dimensions close to the input surface, we aim to minimize the required scaling of the stencil. First, we find the best fitting number of vertices of the stencil. It can be coarsely estimated as $N \approx 0.38A/l_{\text{avg}}^2$ where A is total surface area and $l_{\text{avg}} = 7\,\text{mm}$ is the average unit cell length (using heuristic knowledge that the number of triangles is twice the number of vertices, assuming triangles are close to regular, and approximating unit cell lengths as twice the radius of circumscribed circles). Then, N can be varied to find a value leading to minimal stencil scaling.

Given the layout of bases on the stencil, the bumpers are first constructed as boxes that are aligned along the lines connecting the centers of the triangles and projected onto the corresponding stencil triangle. Matching pairs of boxes are trimmed by the bisector plane between the triangles, defining the interface between neighboring bumpers.

The flat arrangement of bases is then constructed using a minimal distortion conformal map [2] from $\mathcal{T}$ to a resulting 2D mesh, $\mathcal{F}$. We exploit conformal flattening since it circumvents shearing which would result in undesired shear forces in the membrane as it contracts. The mesh $\mathcal{F}$ serves as a stencil for the structure's initial, printed state. We relocate the bases by translation from triangle centers in the final state's stencil to triangle centers in the initial state's stencil. The bases are rotated to align one of its bumper axes in the direction of the corresponding neighbor's center since it is generally not possible to perfectly align all of them. Then, $\mathcal{F}$ is scaled so that the relocated bases with bumpers do not intersect with their neighbors, moreover specifying the minimal gap between them necessary for fabrication (0.1 mm).

Once the flat layout of bases with bumpers is complete, V-shaped brackets that bridge the gaps between bases are generated. This produces a flat structure that fully encodes the target geometry. Conformal flattening may produce overlaps of unit cells, or some of the unit cells may be longer than our upper bound (9.5 mm), making the target shape impossible to replicate. Large unit cell lengths may occur if the target surface has regions with high curvatures or that require substantial stretching during conformal flattening. It is in certain cases possible to resolve the latter issue by placing cuts across the surface [3] or by editing the input geometry. Morphing times are programmed by configuring the thicknesses of the brackets (see the Supplementary Note 4).

OpenSCAD[1] scripts were used to generate STL meshes for the fabricated structures in this study.

Supplementary Note 2: Simulation

Our framework builds on physical simulation to predict the deformed configuration of the system at a given instant in time. We consider a discrete mechanical model that determines the mechanical behavior of a deformable object based on an elastoplastic model with elastic energy potential $W(\mathbf{x}, \bar{\mathbf{x}}(t), \boldsymbol{\kappa}(t)) \in \mathcal{R}$ and plastic energy dissipation formulated through the rest configuration update. Here, $\mathbf{x}(t) \in \mathcal{R}^n$ is a vector containing n generalized coordinates that spatially discretize the kinematic state of the shell in different configurations, $\bar{\mathbf{x}}(t)$ refers to the undeformed configuration, $\boldsymbol{\kappa}(t)$ is a vector grouping all material stiffness parameters, and t is the time instant.

Temporal effects are modeled through the explicit dependencies of the undeformed configuration and material stiffness on time. In the next section, we describe how these dependencies are estimated from empirical data. Because our structures morph at low strain rates, we neglect all dynamic effects and take discrete time increments of constant duration, $\delta = 0.5\,\text{s}$. After each time increment, the undeformed configuration and stiffness parameters are updated quasistatically. We denote the magnitudes corresponding to the k-th time step of the morphing process as $\mathbf{x}^k$, $\bar{\mathbf{x}}^k$, $\boldsymbol{\kappa}^k$. For notation simplicity, we will drop the superscript corresponding to the morphing time step unless specified. Simulating the static behavior of this mechanical system at the k-th morphing time step implies solving the nonlinear system of differential equations defined by net force equilibrium, i.e., $\mathbf{F}(\mathbf{x}^k, \bar{\mathbf{x}}^k, \boldsymbol{\kappa}^k) = -\nabla_{\mathbf{x}} W = \mathbf{0}$, using standard numerical optimization methods.

Our computational model couples a FEM simulation of the membrane, a rigid body model for bases, and a data-driven spring model for the brackets. In the following sections, we describe the kinematics and mechanics of each of these subsystems separately and then specify how we model the coupling between them and solve the numerical problem.

Discrete kinematics

The bases are modeled using N_r prismatic rigid bodies. Each rigid body represents two bases attached to the membrane from the opposite sides. The kinematic state of the i-th rigid body can be determined by the position of the center-of-mass $\mathbf{v}_i \in \mathcal{R}^3$, together with its rotation $\mathbf{r}_i \in \mathcal{R}^3$, expressed in angle-axis format. The corresponding rotation matrix $\mathbf{R}_i$ can be easily computed using the well-known Rodrigues formula

$$\mathbf{R}(\mathbf{r}) = \mathbf{I} + \sin(\theta)[\mathbf{u}]_\times + (1 - \cos(\theta))[\mathbf{u}]_\times^2, \tag{1}$$

where $\theta = ||\mathbf{r}||$, $\mathbf{u} = \mathbf{r}/||\mathbf{r}||$ and $[\mathbf{u}]_\times$ is the cross product matrix of $\mathbf{u}$, i.e., the matrix such that $[\mathbf{u}]_\times \mathbf{x} = \mathbf{u} \times \mathbf{x}$. This allows us to express the position of any point $\mathbf{p}_j$ in the local coordinates of the i-th rigid body through the non-linear relation $\mathbf{p}_j = \mathbf{R}_i(\mathbf{r}_i)\mathbf{p}_{ji}^0 + \mathbf{v}_i^k$, where $\mathbf{p}_{ji}^0 = \mathbf{p}_j^0 - \mathbf{v}_i^0$, are the coordinates of the point in the local frame of the rigid body.

Each pair of neighboring bases are joined by two brackets. The N_s brackets are modeled using two types of components: data-driven springs and shear-resisting elements. Both components are composed by line segments denoted $\mathbf{s}_{ij} = (\mathbf{s}_i, \mathbf{s}_j)$, where $\mathbf{s}_i$ and $\mathbf{s}_j$ are a pair of points on the surface of the i-th and j-th bases.

- Data-driven springs (Supplementary Figure 3, left), $\mathbf{c}_{ij}^q$, for $q = 1, \ldots, 4$, are responsible for modeling the time-evolving resistance to deformation as well as bumper collisions.

- Shear-resisting elements (Supplementary Figure 3, center), represented by crossing pairs of segments $\mathbf{s}_{ij}^q = \{\mathbf{s}_{ij}^{qa}, \mathbf{s}_{ij}^{qb}\}$, for $q = 1, \ldots, 4$, are responsible for penalizing undesired in-plane and out-of-plane shearing during the simulation. Resistance to shearing is inherent to the fabricated brackets due to their V-shaped design.

Finally, we represent the elastic membrane as a piecewise linear mesh of triangles (Supplementary Figure 3, right), with N_m vertices. The set of membrane vertices $\mathcal{M} = \{\mathbf{m}_1, \ldots, \mathbf{m}_{N_m}\}$ can be partitioned into two subsets: free vertices, $\mathcal{M}_\text{f} = \{\mathbf{f}_1, \ldots, \mathbf{f}_{N_\text{f}}\}$, and vertices coupled to the bases, $\mathcal{M}_\text{g} = \{\mathbf{g}_1, \ldots, \mathbf{g}_{N_\text{g}}\}$.

[1] http://www.openscad.org

All points lying on the surface of a base are coupled to it implicitly. The positions of these points can be expressed in terms of the center-of-mass and rotation of the rigid bodies following the non-linear expression in the Supplementary Equation 1. Therefore, the geometric configuration of the shell can be completely determined by the positions and orientations of the rigid bodies together with the membrane vertices that are not coupled to the rigid bodies. This leads to a total of $N_\mathrm{t} = 6N_\mathrm{r} + 3N_\mathrm{f}$ degrees of freedom which we group in the state vector $\mathbf{x} = \{\mathbf{v}_1, \mathbf{r}_1, \ldots, \mathbf{v}_{N_\mathrm{r}}, \mathbf{r}_{N_\mathrm{r}}, \mathbf{f}_1, \ldots, \mathbf{f}_{N_\mathrm{f}}\}$.

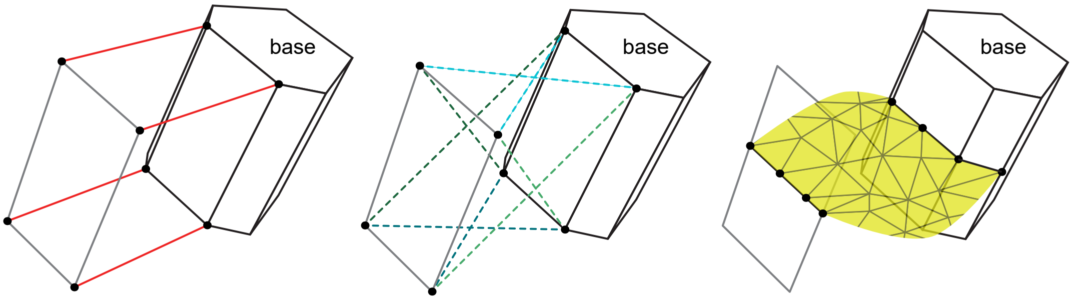

Supplementary Figure 3: Discretization elements: data-driven springs, representing brackets' time-evolving stiffness and bumper collisions (left); shear-resisting elements, representing brackets' resistance to undesired shearing (center); and membrane FEM (right).

Discrete energies

Given this discretization, the mechanical behavior of the shell can be described by a conservative elastic potential W aggregating the contributions of the data-driven springs W_c, shear-resisting energy W_s, and the membrane W_m:

$$W(\mathbf{x}) = \sum_{U_{ij}} \left(\sum_{q=1}^{4} W_\mathrm{c}(\mathbf{c}_{ij}^{q}) + \sum_{q=1}^{4} W_\mathrm{s}(\mathbf{s}_{ij}^{qa}, \mathbf{s}_{ij}^{qb}) \right) + \sum_{\mathcal{T}_i} W_\mathrm{m}\big(\mathbf{G}_i(\mathbf{m}_{\mathcal{T}_i})\big). \tag{2}$$

here, U_{ij} refers to the unit cell joining the i-th and the j-th bases, $\mathcal{T}_i$ refers to the i-th element of the membrane discretization, and $\mathbf{G}_i$ is the deformation gradient of the membrane evaluated at this element. Let us separately explain each of the energy terms:

- The data-driven spring energy W_c, has the following expression:

$$W_\mathrm{c}(\mathbf{c}_{ij}^{q}) = \begin{cases} W_\mathrm{d}\Big(b, h, t, \bar{L} - L(\mathbf{c}_{ij}^{q})\Big) & \text{if} \quad L(\mathbf{c}_{ij}^{q}) > L_\mathrm{c}, \\ \frac{\kappa_\mathrm{c}}{2}\Big(L(\mathbf{c}_{ij}^{q}) - L_\mathrm{c}\Big)^2 & \text{if} \quad L(\mathbf{c}_{ij}^{q}) \le L_\mathrm{c}, \end{cases} \tag{3}$$

  where L_c is the collision distance for the spring determined by the bumpers geometry. Initially, the elastoplastic behavior of the spring follows a data-driven model $W_\mathrm{d}(b, h, t, x)$ for a given bracket length, b, bracket thickness, h, and time spent under water, t (see next section). It depends on spring deformation, $x = \bar{L} - L(\mathbf{s}_{ij}^{q})$, where $\bar{L}$ is the rest length of the spring. To account for plastic effects, the rest length of the spring is updated after each time increment following the scheme $\bar{L}^{k+1} = \min\Big(\bar{L}^k, \bar{L}^0 - \eta(\bar{L}^0 - L^k)\Big)$ with constant plasticity fraction η. Once the current length of the spring is smaller than the collision distance, the actuation is stopped and the collision distance is enforced using a soft constraint defined through a high stiffness constant, κ_c.

- The shear-resisting energy W_s, has the following expression:

$$W_\mathrm{s}(\mathbf{s}_{ij}^{qa}, \mathbf{s}_{ij}^{qb}) = \frac{\kappa_s}{2}\Big(L(\mathbf{s}_{ij}^{qa}) - L(\mathbf{s}_{ij}^{qb}) - R^0\Big)^2, \tag{4}$$

  where $L(\mathbf{s}_{ij}) = \|\mathbf{s}_i - \mathbf{s}_j\|$, is the distance between the spring segment end points, R^0 is the difference between distances in the initial morphing time step, and κ_s is a constant.

- For the membrane energy W_m, we use a classical FEM formulation with an incompressible Neo-Hookean material [4]. Continuum magnitudes are interpolated from nodal values using linear basis functions which allows us to discretely approximate the deformation gradient $\mathbf{G} = \nabla_{\bar{\mathbf{m}}}\mathbf{m}$. Here, the undeformed configuration can be computed from the membrane state at the initial configuration $\bar{\mathbf{m}} = \tau^{-1}\mathbf{m}^0$, where τ is the pre-stretch factor.

Coupling and solver

At each time step of the morphing process, we formulate and solve the nonlinear system of differential equations defined by the net force equilibrium $-\nabla_\mathbf{x} W = \mathbf{0}$, by minimizing the discrete elastic potential in the Supplementary Equation 2. We solve this problem using Newton-Raphson method with Strong Wolfe convergence conditions for step length selection.

Solving this problem efficiently requires analytically computing both the first $\nabla_\mathbf{x} W$ and second $\nabla^2_{\mathbf{xx}} W$ derivatives of the elastic potential. As introduced above, points lying on the surface of the bases, $\mathbf{p}$, are implicitly coupled to the rigid bodies through the Supplementary Equation 1. Hence, these derivatives can be easily computed using the chain-rule:

$$\frac{\partial W}{\partial \mathbf{x}} = \frac{\partial W}{\partial \mathbf{f}}\frac{\partial \mathbf{f}}{\partial \mathbf{x}} + \frac{\partial W}{\partial \mathbf{p}}\frac{\partial \mathbf{p}}{\partial \mathbf{x}}, \qquad \frac{\partial^2 W}{\partial \mathbf{x}^2} = \frac{\partial \mathbf{f}}{\partial \mathbf{x}}^T \frac{\partial^2 W}{\partial \mathbf{f}^2}\frac{\partial \mathbf{f}}{\partial \mathbf{x}} + \frac{\partial \mathbf{p}}{\partial \mathbf{x}}^T \frac{\partial^2 W}{\partial \mathbf{p}^2}\frac{\partial \mathbf{p}}{\partial \mathbf{x}}\frac{\partial W}{\partial \mathbf{p}^2}\frac{\partial^2 \mathbf{p}}{\partial \mathbf{x}^2}, \tag{5}$$

where $\nabla_\mathbf{x}\mathbf{f}$ is a selection matrix of the free membrane vertices and $\nabla_\mathbf{x}\mathbf{p}$ and $\nabla^2_{\mathbf{xx}}\mathbf{p}$ can be computed from the Supplementary Equation 1. One common technique in rigid-body simulation to simplify this computation is to keep rotational degrees of freedom $\mathbf{r}_i$ close to zero. This is done by updating the local coordinates of the coupled points in the base frame after each successful iteration, i.e., for the j-th point attached to the i-th rigid body, $\mathbf{p}^0_{ji} \leftarrow \mathbf{R}(\mathbf{r}_i)\mathbf{p}^0_{ji}$, $\mathbf{r}_i \leftarrow \mathbf{0}$.

We provide the following data as an example of the simulation scales. Our most complex model with self-interweaving shape (Fig. 3d) contains 549 rigid bodies and 7942 membrane elements. The full morphing process simulation (240 time increments) takes 43 minutes in total.

Supplementary Note 3: Material measurement and modeling

As discussed in the Supplementary Note 2, we represent brackets in simulations by data-driven springs and shear-resisting elements. Here we describe our approach to the mechanical modeling of the data-driven components. The brackets in our structure undergo large deformations and are made of a material with nonlinear elastic properties and time-dependent softening. This combination of material and geometric nonlinearities leads us to a data-driven effective spring model. We performed all measurements in settings that resemble conditions brackets are subjected to in an assembled structure. We first formulate the data-driven elastoplastic spring model $W_\mathrm{d}(b, h, t, x)$ (introduced in the previous section) and then describe our fitting strategy. We discuss several polynomial fittings in this section and display their output units in square brackets.

Physical model of brackets

Our elastoplastic bracket model is motivated by a set of material tests described below. The elastic component is expressed through an effective stiffness, and the plastic behavior is described as a dissipation of internal elastic energy due to deformation. We make the approximation of assuming a constant plasticity fraction, η, which we obtained experimentally. The plastic part of the displacement is then given by $x_\mathrm{pl} = \eta x$ and the elastic part is $x_\mathrm{el} = (1-\eta)x$.

We aim to obtain elastic energy formulations $W_\mathrm{d}(b, h, t, x_\mathrm{el})$ that are functions of time t spent in hot water for each valid combination of bracket length b and thickness h. These formulations are modelled as trilinear interpolations between polynomials $p_{b,h,t}(x_\mathrm{el})$ that are defined on a regular grid.

Under the assumption of a constant plasticity fraction and with material properties corresponding to time t, we relate elastic energy $W_\mathrm{d}(b, h, t, x_\mathrm{el})$ to the total external work $\bar{W}$ done through monotonic bracket

displacements x in the following manner:

$$W_\mathrm{d}(b,h,t,x_\mathrm{el}) = (1-\eta)\bar{W}\Big(b,h,t,\frac{x_\mathrm{el}}{1-\eta}\Big). \tag{6}$$

Here, $\bar{W}$ are trilinear interpolations of polynomials $p^\mathrm{work}_{b,h,t}(x)$ [Nm] that are similarly related to $p_{b,h,t}(x_\mathrm{el})$:

$$p_{b,h,t}(x_\mathrm{el}) = (1-\eta)p^\mathrm{work}_{b,h,t}\Big(\frac{x_\mathrm{el}}{1-\eta}\Big). \tag{7}$$

These polynomials are obtained by integrating force over displacement:

$$p^\mathrm{work}_{b,h,t}(x) = \int_0^x p^\mathrm{load}_{b,h,t}(\tilde{x})d\tilde{x}\,, \tag{8}$$

where $p^\mathrm{load}_{b,h,t}(x)$ [N] are polynomials representing the loads exerted on bracket over applied displacements. The polynomials $p^\mathrm{load}_{b,h,t}(x)$ are fourth-order in x with no free term. The fitting methods for obtaining these polynomials are discussed in the following subsection.

It is challenging to measure time-dependent force-displacement relationships directly in an experimental setup since specimen submersion and loading takes a significant amount of time relative to our actuation time ranges. Specimen submersion and loading takes 8 seconds while the target deformations last approximately 30 to 80 seconds. This restrains us from an assumption that material properties are "fixed" at time t for the material measurements. Additionally, we collected a higher density of data in time rather than in displacement due to the capabilities of our experimental setup. This leads us to dividing the fitting problem into simpler components. We first fit (inverted) displacement-force relationships and then use the obtained model to reconstruct the desired force-displacement model $p^\mathrm{load}_{b,h,t}(x)$. Our displacement-force model is the following:

$$x(b,h,t,F) = p^\mathrm{dry}(b,h,F) + \int_0^t F\exp\big(p^\mathrm{wet}(b,h,\tilde{t},F)\big)d\tilde{t}, \tag{9}$$

where p^dry [m] is a third-order polynomial (limited to first order in b and h) for which all terms have F as a multiplier and p^wet [$\log\left(\mathsf{mN^{-1}s^{-1}}\right)$] is a fourth-order polynomial. Polynomial p^dry represents displacement-force relationships before putting into water while p^wet is the logarithmic evolution of deformation rates, divided by load, in water. The latter formulation restricts the deformation speed to be always positive which is a desirable property for our model and assures zero displacement under zero load. The integral does not generally have a simple explicit representation.

We apply the transformation mentioned above to the measured deformation speeds and fit the resulting data

$$p^\mathrm{wet}(b,h,t,F) = \log\left(F^{-1}\frac{\partial x}{\partial t}\right). \tag{10}$$

The logarithmic function improves the quality of fitting by reducing extreme variation in deformation rates. Note that the formulation of displacement-force relationships is defined as a single continuous function across all parameters in our setup. This allows us to build a consistent model of data collected from all experimental measurements.

Data collection and fitting

A Zwick tensile tester (shown in the Supplementary Figure 4c) was used for all bracket characterization experiments. The specimens tested (shown in the Supplementary Figure 4a) were attached to a pair of prismatic grippers (Supplementary Figure 4b) to enforce uniaxial movement.

Three types of experiments were conducted: compression tests in both dry and wet states, as well as effective plasticity measurements. For the dry compression tests, displacement-force relations for brackets that hadn't been immersed in water were obtained quasistatically. For the wet compression tests, we immersed specimens into hot water and immediately applied constant loads. These specimens deformed gradually over time since exposure to hot water causes them to soften. For the effective plasticity tests, we compressed brackets by a prescribed displacement, unloaded them, and measured the restoration to derive the plastic component of the deformation. For all tests we used the same sampling of central lengths l (from the

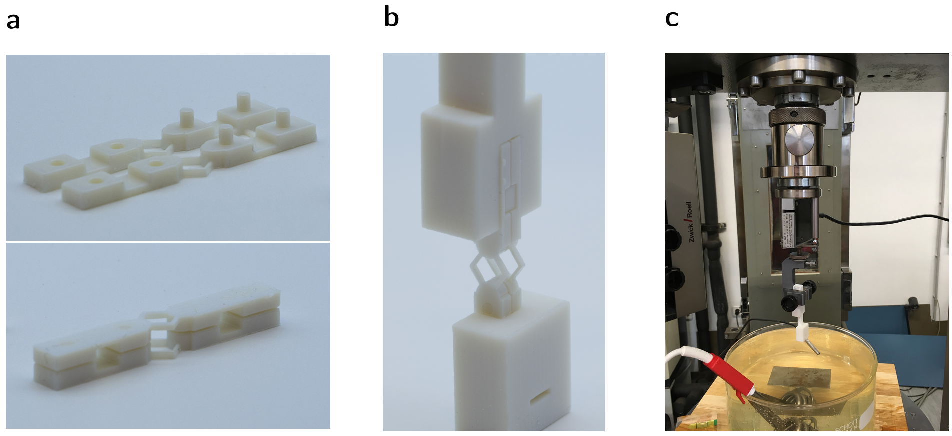

Supplementary Figure 4: (a) Specimens used for material measurements are assembled from two printed parts to mimic a unit cell. Assembled specimens have holes to ensure consistent boundary conditions in a gripper that was fabricated in-house. (b) Custom-built gripper for quick specimen exchange and a "boot" for firm specimen compression against the floor. (c) Zwick tensile tester for measuring bracket deformations in hot water.

Supplementary Figure 1, it is easy to see that $l = b + 4\cos 37°)$ and bracket thicknesses h (in millimeters): $l \in \{5, 6, 7, 8, 9\}$, for $l \leq 7$ we choose $h \in \{0.3, 0.4, 0.5, 0.6\}$ and for $j > 7$ we choose $h \in \{0.35, 0.45, 0.55, 0.65\}$. This set of parameters amounts to 20 total combinations.

Fitting polynomials with constrained derivatives

Empirical knowledge (such as the fact that thicker brackets deform at slower rates) was used to derive constraints on fitted functions. Similar to standard polynomial regression, we solve a quadratic programming problem $y = X\beta + \epsilon$, but instead of $\beta = (X^T X)^{-1} X^T y$ we solve:

$$\begin{array}{ll} \texttt{minimize} & \frac{1}{2}\beta^T X^T X\beta - y^T X\beta \\ \texttt{subject to} & A\beta \leq 0\,, \end{array} \tag{11}$$

where A expresses row-wise derivative constraints per point. By providing sufficiently many points in A we significantly improve fitted curve quality. We impose these constraints at all given data points. All the constraints used for each polynomial fitting are listed below.

Compressive loading of dry specimens

This experiment is conducted once for each specimen by increasing the applied load quasi-statically from 0 to 10 N. We fit to this data a polynomial p^{dry} expressing resulting displacements x given the loading F. Note that displacement is a monotonic function of bracket length b, unlike strain. We impose the following derivative constraints:

$$\frac{\partial p^{\text{dry}}}{\partial b} > 0, \quad \frac{\partial p^{\text{dry}}}{\partial h} < 0, \quad \frac{\partial p^{\text{dry}}}{\partial F} > 0. \tag{12}$$

The resulting curves are in the Supplementary Figure 5.

Compressive loading of specimens in water

The next step is to model the evolution of the displacement-force curves over time spent in water. In order to do so, we test the behavior of each specimen under various loads (which are held constant throughout an individual test).

Since the initial force-displacement relationships p^{dry} are known with relatively high precision, we use those curves as our model for $t = 0$. We fit the displacement rate data to reconstruct the time-evolution of bracket behavior in water. We impose the following constraints on the derivatives:

$$\frac{\partial p^{\text{wet}}}{\partial b} > 0, \quad \frac{\partial p^{\text{wet}}}{\partial h} < 0, \quad \frac{\partial p^{\text{wet}}}{\partial t} > 0, \quad \text{and} \quad \frac{\partial p^{\text{wet}}}{\partial F} > 0. \tag{13}$$

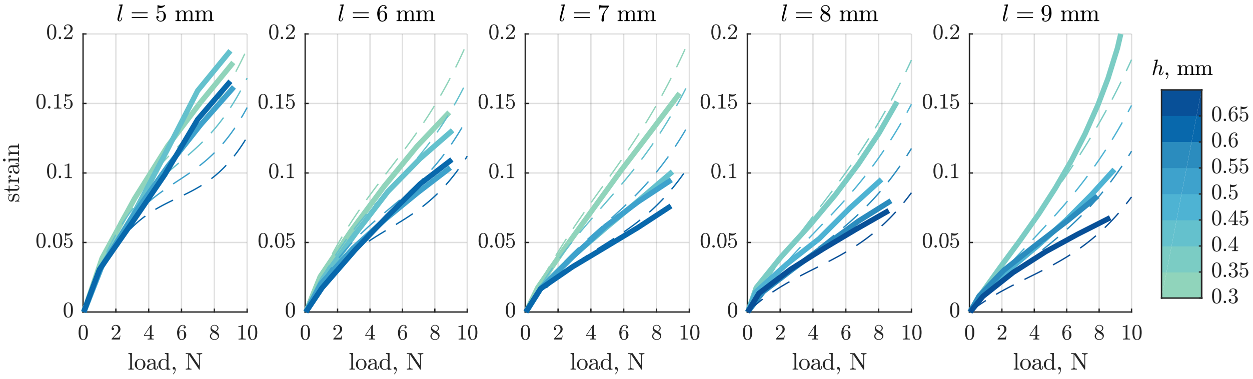

Supplementary Figure 5: Compressive loading of dry specimens. Data (solid lines) and fitted curves (dashed lines).

Then we reconstruct time-evolving displacement-force relationships in the Supplementary Equation 9. The resulting curves are shown in the Supplementary Figure 6. We filter out displacements larger than $^2/_3$ of the initial length since brackets may be damaged and display inconsistent behavior at that point.

After obtaining a displacement-force relationship, we invert it with respect to displacement. We densely resample our target parameter space of b, h, t and refit force-displacement curves represented by polynomials $p^{\text{load}}_{b,h,t}(x)$ with constrained derivatives:

$$\frac{\partial p^{\text{load}}_{b,h,t}(x)}{\partial x} > 0 \quad \text{and} \quad \frac{\partial^2 p^{\text{load}}_{b,h,t}(x)}{\partial x^2} < 0. \tag{14}$$

Effective plasticity tests

In order to properly simulate actuation of the structure, we examine elastic energy dissipation during bracket compression. We approximate this phenomenon through an elasto-plastic model. We do not study the dependence of plasticity on temperature because all experiments occur at 56 ^oC. Thus, we analyze the dependence of plasticity on deformation rates. This experiment is done for specimens with fixed chosen parameters $l = 8\,\text{mm}$ and $h = 0.45\,\text{mm}$. We do multiple loading tests with different deformation rates and measure the restored strain after unloading. We observed that there is no significant dependence on deformation rates (see the Supplementary Figure 7). We did not observe consistent strain restoration across different bracket thicknesses and lengths, with mean plastic fractions in a range 15%–25%. We use a constant mean plasticity fraction $\eta = 20\%$ in our simulations.

Supplementary Note 4: Temporal programming

In our design pipeline, the user specifies a time landscape. This is a smooth scalar field over the target surface that represents the desired deformation completion time at each point. In our implementation we define it as a piecewise linear function on top of the flat stencil triangulation. For each unit cell, we compute the average desired actuation time, t^*, using values specified at the ends of the associated stencil edge. Then we use t^* to configure the thicknesses of the brackets associated with the unit cell. This computation is based on the effective force-displacement curves modelled by trilinear interpolation of polynomials $p^{\text{load}}_{b,h,t}(x)$, which we denote as $F(b, h, t, x)$. Each unit cell's deformation is consistent with its neighbors' so long as the time landscape is sufficiently smooth.

Both pairs of brackets on the opposite sides of the unit cell have the same initial length b_0 by construction. We first compute their length in the fully actuated state b_1 (in general, different for different sides). Then, for the target actuation time t^*, we can formulate the bracket thickness h configuration problem as follows:

$$F(b_0, h, t^*, b_0 - b_1) = F^{\text{mem}}(b_0, d_1), \tag{15}$$

where F^{mem} is an approximation of the traction generated by the membrane dependent on the initial unit cell length, b_0, and on a parameter describing membrane deformation, d_1 with its value at the actuated state.

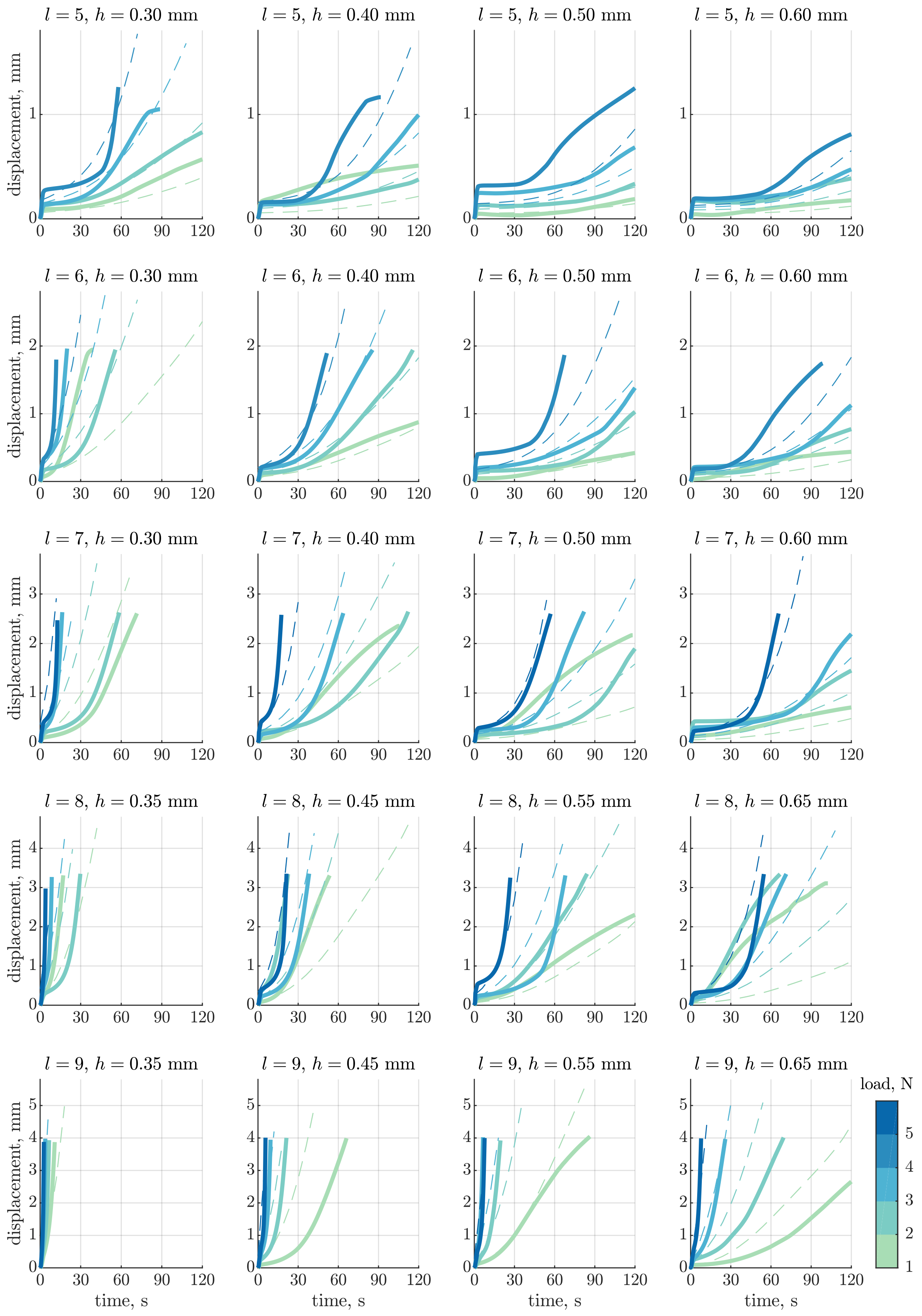

Supplementary Figure 6: Compressive loading of specimens in water. Data (solid lines) and fitted curves (dashed lines).

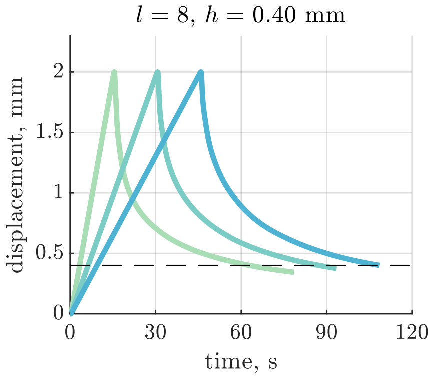

Supplementary Figure 7: Plasticity does not depend on deformation rates. Three different deformation rates are shown for a unit cell specimen of length $l = 8\,\text{mm}$ and thickness $h = 0.4\,\text{mm}$. Dashed line represents 20% of maximal deformation which we use as a constant plasticity fraction in our simulations.

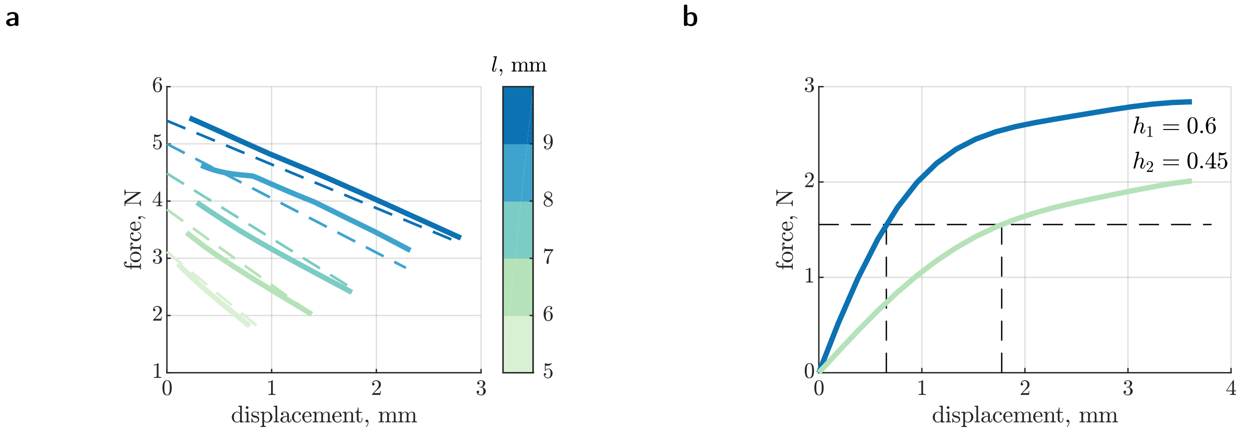

Supplementary Figure 8: (a) Linearized model of the membrane (dashed lines) in comparison to FEM membrane (solid lines) for a set of unit cells of various initial lengths. The membrane tractions decrease with displacement as the pre-stretch is relaxed. (b) Configuring thicknesses for two pairs of brackets on opposite sides of a unit cell. Note that the one requiring larger target displacement is thinner to finish deformation at the same time as the one with smaller target deformation. The dashed horizontal line shows a sample approximation to the target membrane traction.

Here we use the force balance between the brackets and the membrane at the target deformation after time t^* spent in water, ignoring plasticity (Supplementary Figure 8b).

We build a linearized model of the membrane, representing it as a segment spring connecting the centroids of the end points of all brackets in a unit cell, and setting $F^{\text{mem}}(b, d)$ as a first-order polynomial in each of the variables. Here b mimics an initial membrane spring length and d is its displacement. We fit this model by sampling initial bracket lengths and deformations. It captures membrane forces well in our setting (Supplementary Figure 8a).

Our approach to finding the thickness h is a binary search through our interpolated model of $F(b_0, h, t^*, b_0 - b_1)$ to match a known value of $F^{\text{mem}}(b, d)$.

We need a specific treatment of boundary unit cells since the membrane ends there and has less stretch initially. To account for this, we reduce the force evaluation by a factor of 0.6 which was empirically found by comparing simulations of our examples with the membrane represented by linear springs to FEM membrane simulations.

Once the desired time landscape is specified and bracket thicknesses are computed accordingly, we start the simulation process described in the Supplementary Note 2. Unit cells' actuation completion might deviate from the specified time landscape due to imperfections in the linearized membrane force estimation and plastic effects. Apart from that, the resulting morphing process might not reach the final goal, for example there might be collisions on the way. In these cases time landscape has to be edited and the

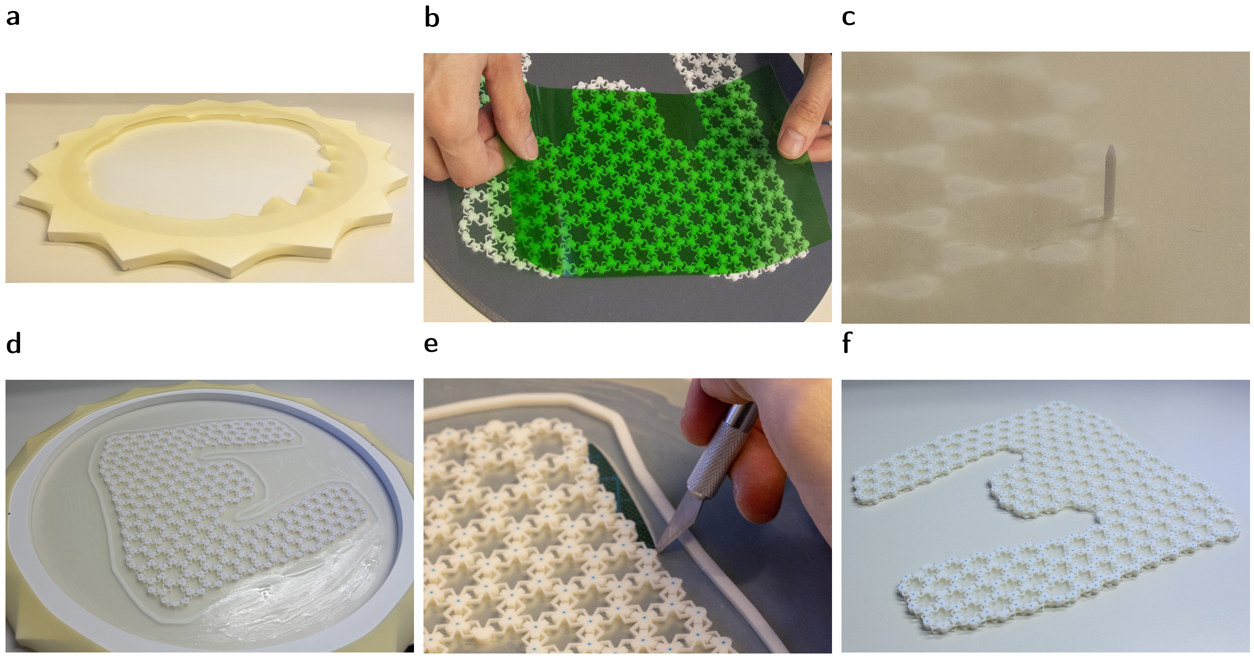

Supplementary Figure 9: Fabrication process landmarks. (a) Star-shaped membrane stretching device back side up. Bottom part of the membrane is uniformly stretched due to markers. (b) Transferring glue from a plastic foil to the bases of the shell. (c) Passing a pin through one of the bases and the membrane to align with the second lattice. (d) Membrane surplus is covered by glue in order to "freeze" it and enable its easy removal. (e) Cutting out the shell from the membrane surplus by a scalpel. (f) Flat-fabricated shell ready for actuation in water.

simulation of morphing has to be recomputed until the goal is achieved.

Supplementary Note 5: Fabrication procedure

We developed a custom fabrication procedure for the shells. It is described in detail in this section.

1. 3D print the outer layers of the shells using a Stratasys J750 printer. We also printed an outer frame to reduce undesired deformation of the structure during fabrication. Printing time for all of our shells is approximately 2 hours (it mostly depends on the number of layers, which is fixed). It is twice as long for the first petalled shape (Fig. 1c) since both sides do not fit on a single printing tray. We remove all overhangs, which does not affect shape replication but makes the cleaning process drastically easier: we only quickly remove almost all support material by several shaving movements with a sharp scraper and airflow the lattice to erase the rest.

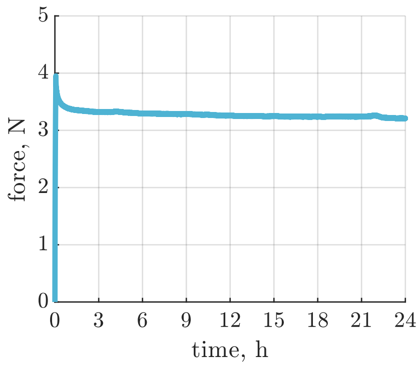

Supplementary Figure 10: Membrane stress relaxation over the course of 24 hours. Evolution of the force generated by a dog-bone membrane specimen under a constant stretch factor of 3.

2. Use a star-shaped device to manually stretch a latex sheet uniformly by gradually wrapping it around and fixing at the device's teeth (Supplementary Figure 9a). Friction between the membrane and the device is sufficient to keep the membrane stretched without any additional clamps. A stretch factor of 3 (900% the area) is enforced by matching markers on the sheet to the tips of the device's teeth. Note that large deformations of the membrane lead to slight stress relaxation in the long run. Given the variability of the rest of the system and our simplified membrane model, we neglect this effect. It is however possible to take it into account since after approximately 1.5 hours stress relaxation does not progress much further (Supplementary Figure 10).

3. Clean both surfaces of the sheet with 2-Propanol for better gluing. Apply super glue on a plastic film and distribute it uniformly with a brush. Transfer glue from the film to the 3D printed structures through contact with the plastic film (Supplementary Figure 9b).

4. Glue one lattice to the membrane and pass push pins through the alignment holes on the lattice and through the latex membrane (Supplementary Figure 9c). The membrane does not rip since we pinch it through an isolated area.

5. Glue the opposite side to the latex sheet matching the holes with corresponding alignment pins.

6. Distribute additional super glue on the latex membrane in the region surrounding the lattice perimeter (Supplementary Figure 9d).

7. Wait 5 minutes and then release the latex sheet to a stretch factor near 2. Then using a scalpel cut out the structure, keeping a tiny amount of degenerated latex at the border to prevent membrane ripping (Supplementary Figure 9e).

8. Submerge the structure in 56° C water and wait for morphing to complete.

9. Take out the deformed shape and let it dry. Under normal conditions the drying process may take roughly 15 minutes.

The whole fabrication process after 3D printing and before drying takes 30–50 minutes.

Supplementary Note 6: Mechanical measurements of shells

We performed a set of mechanical measurements of the shells for estimation of their load-bearing capabilities. We fabricated rectangular and flat regularly tessellated shells composed of identical unit cells of length 7 mm, bracket thickness 0.5 mm, and bumpers 0.3 mm with total dimensions 60×55 mm. Since the shell structure is not isotropic, we performed the tests aligning the deformation to one of the three axes parallel to the edges of the hexagonal pattern. The resulting plots are shown in the Supplementary Figure 11. In all tests the deformation speed was approximately 1 mm/min. It is intuitive that the shells have higher resistance to pure in-plane compression due to the bumper contacts in contrast to the other cases when only the membrane and the brackets are loaded.

References

1. Liu, Y. *et al.* On Centroidal Voronoi Tessellation–Energy Smoothness and Fast Computation. *ACM Trans. Graph.* **28,** Article 101 (Aug. 2009).
2. Springborn, B., Schröder, P. & Pinkall, U. Conformal Equivalence of Triangle Meshes. *ACM Trans. Graph.* **27,** 77:1–77:11 (Aug. 2008).
3. Guseinov, R., Miguel, E. & Bickel, B. CurveUps: Shaping Objects from Flat Plates with Tension-actuated Curvature. *ACM Trans. Graph.* **36,** 64 (2017).
4. Ogden, R. D. *Non-linear Elastic Deformations* (Dover Publications, 1997).

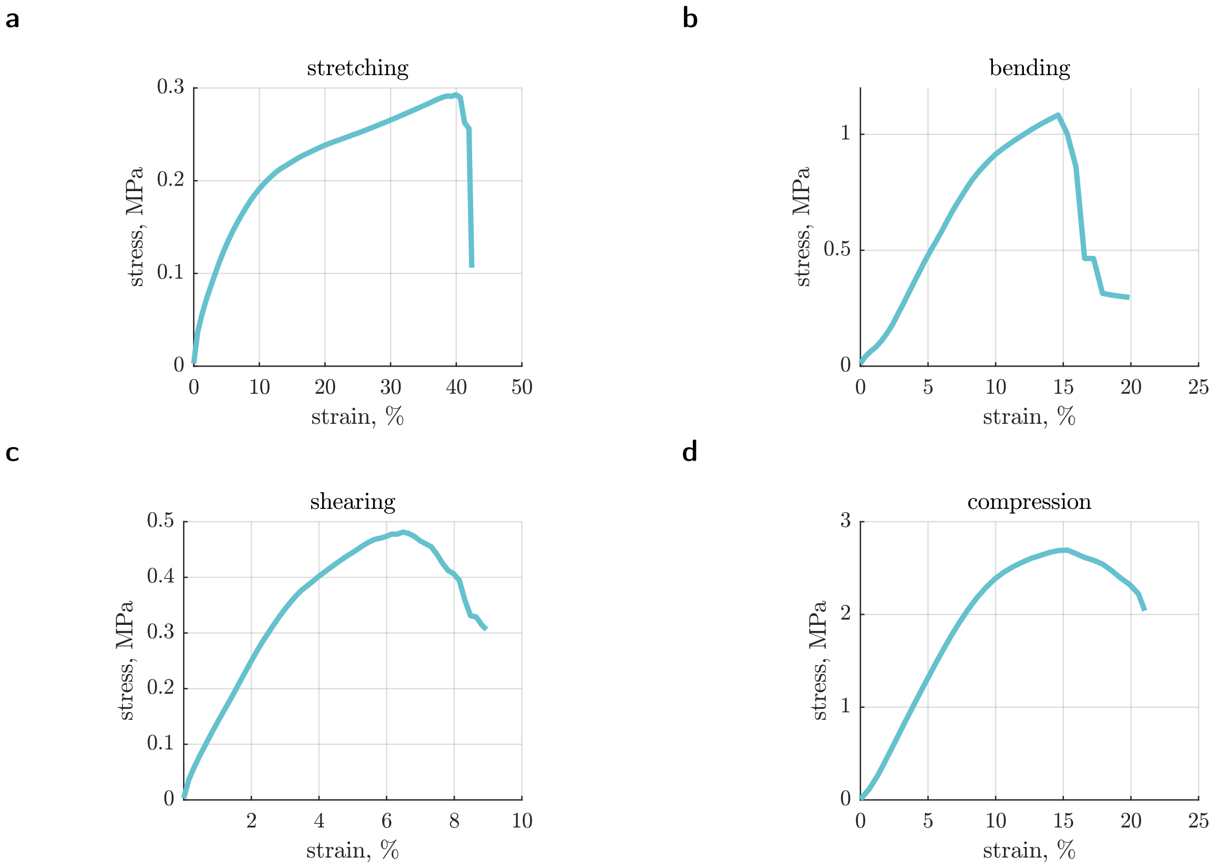

Supplementary Figure 11: Mechanical tests of a flat regularly tessellated shell. Since our shells have cross-sections with a complex geometry, we provide the effective stress values (assuming shell homogeneity). (a) Stretching, (b) bending, (c) shearing, and (d) compression tests.
