# Supplementary material for: Programming temporal morphing of self-actuated shells
Source: Nat Commun. 2020 Jan 13;11:237. doi: 10.1038/s41467-019-14015-2 (PMC6957700; doi:10.1038/s41467-019-14015-2)
Supplement: Supplementary file 3 — Description of Additional Supplementary Files [file 41467_2019_14015_MOESM3_ESM.pdf]

Description of Additional Supplementary Files

Supplementary Movie 1. Morphing process of the petalled structure 1.

Supplementary Movie 2. Morphing process of the petalled structure 2.

Supplementary Movie 3. Morphing process of a double-loop spiral.

Supplementary Movie 4. Morphing process of a saddle.

Supplementary Movie 5. Morphing process of a self-interweaving shape.
