# Supplementary material for: Programming temporal morphing of self-actuated shells
Source: Nat Commun. 2020 Jan 13;11:237. doi: 10.1038/s41467-019-14015-2 (PMC6957700; doi:10.1038/s41467-019-14015-2)
Supplement: Supplementary file 2 — Peer Review File [file 41467_2019_14015_MOESM2_ESM.pdf]

Editorial Note: This manuscript has been previously reviewed at another journal that is not operating a transparent peer review scheme. This document only contains reviewer comments and rebuttal letters for versions considered at Nature Communications.

Reviewers' Comments:

Reviewer #1:
Remarks to the Author:
The authors have made all my suggested changes and thus I believe the manuscript is definitely publication worthy - particularly for Nature Communications.

Point-by-point response to issues raised by the referees

Reviewer #3 (Remarks to the Author):

The authors have made all my suggested changes and thus I believe the manuscript is definitely publication worthy - particularly for Nature Communications.

We thank the reviewer for their positive feedback.

Sincerely,

Chiara Daraio,
Caltech
daraio@caltech.edu

and

Bernd Bickel,
IST Austria
bernd.bickel@ist.ac.at
